# AMPK stimulation inhibits YAP/TAZ signaling to ameliorate hepatic fibrosis

**DOI:** 10.1038/s41598-024-55764-5

**Published:** 2024-03-03

**Authors:** Mahbubul H. Shihan, Sachin Sharma, Carson Cable, Vijaya Prathigudupu, Alina Chen, Aras N. Mattis, Jennifer Y. Chen

**Affiliations:** 1 Department of Medicine, University of California, San Francisco, San Francisco, CA 94115 USA; 2 Department of Pathology, University of California, San Francisco, San Francisco, CA 94143 USA; 3 The Liver Center, Department of Medicine, University of California, San Francisco, San Francisco, CA 94143 USA

**Keywords:** Molecular biology, Diseases, Gastroenterology, Molecular medicine, Pathogenesis

## Abstract

Hepatic fibrosis is driven by the activation of hepatic stellate cells (HSCs). The Hippo pathway and its effectors, YAP and TAZ, are key regulators of HSC activation and fibrosis. However, there is a lack of mechanistic understanding of YAP/TAZ regulation in HSCs. Here we show that AMPK activation leads to YAP/TAZ inhibition and HSC inactivation in vitro, while the expression of a kinase-inactive mutant reversed these effects compared to wild type AMPKɑ1. Notably, the depletion of LATS1/2, an upstream kinase of YAP/TAZ signaling, rescues YAP/TAZ activation, suggesting that AMPK may be mediating YAP/TAZ inhibition via LATS1/2. In the carbon tetrachloride mouse model of fibrosis, pharmacologic activation of AMPK in HSCs inhibits YAP/TAZ signaling and reduces fibrosis. The findings implicate AMPK as a critical regulator of YAP/TAZ signaling and HSC inactivation and highlight AMPK activation as a therapeutic target for the treatment of hepatic fibrosis.

## Introduction

Cirrhosis, or end-stage liver disease, is the 14th leading cause of morbidity and 11th leading cause of death globally^1^. While liver transplantation remains an effective therapy for patients with cirrhosis, it is limited by organ availability. Hepatic fibrosis is the common pathological mechanism that leads to cirrhosis and occurs following chronic liver injury from toxins (alcohol), infections (hepatitis B and hepatitis C), and metabolic dysfunction-associated steatotic liver disease (MASLD). Fibrosis is driven by hepatic stellate cells (HSCs). In response to chronic injury, HSCs undergo activation, leading to deposition and cross-linking of extracellular matrix proteins that result in the fibrotic scar.

It has been demonstrated that hepatic fibrosis is reversible in some patients: for example, successful treatment of HCV infection has resulted in fibrosis regression and improvement in health outcomes^2^. Moreover, preclinical studies have shown that HSCs revert to an inactivated phenotype during fibrosis regression^3,4^, which suggests that targeting HSC inactivation may promote regression in patients with chronic liver disease.

The Hippo pathway and its effectors, YAP and TAZ, are key regulators of HSC activation and hepatic fibrosis: we and others have shown that YAP drives hepatic stellate cell (HSC) activation, and YAP inhibition leads to HSC inactivation and fibrosis regression^5–10^. These studies highlight YAP/TAZ as a core signaling cascade in hepatic fibrogenesis. However, direct inhibition of YAP/TAZ signaling for the treatment of fibrosis may have several limitations. YAP/TAZ are ubiquitously expressed, and studies have demonstrated that the function of YAP/TAZ signaling is complex, disease-specific, and cell-type dependent^11^. For example, studies have demonstrated that YAP/TAZ activation is crucial to improving liver regeneration^12,13^. In hepatocarcinogenesis, YAP activation represents an early event, and pharmacologic inhibition of YAP signaling attenuates disease progression^14,15^. Others have described that YAP/TAZ activation in normal peritumoral tissue suppresses tumor growth^16^. Thus, systemic inhibition of YAP/TAZ may have adverse effects depending on the disease context and cell targets.

Consequently, it is important to elucidate HSC-specific signals that modulate YAP/TAZ activity to reduce possible undesired effects for the treatment of patients with fibrosis. Currently, the knowledge regarding the mechanistic regulation of YAP/TAZ signaling in HSCs is limited. In this study, we aimed to elucidate regulators of YAP/TAZ signaling in HSCs. Here, we identify AMPK, a key cellular energy sensor that modulates cellular metabolism^17^, as a suppressor of YAP/TAZ signaling in HSCs. We demonstrate using cell culture and a mouse model of fibrosis the relationship between AMPK, YAP/TAZ, and HSC activation. This work reveals molecular insights of YAP regulation in HSCs and hepatic fibrogenesis.

## Results

### Energy stress and YAP/TAZ signaling in HSCs

**Figure 1:**
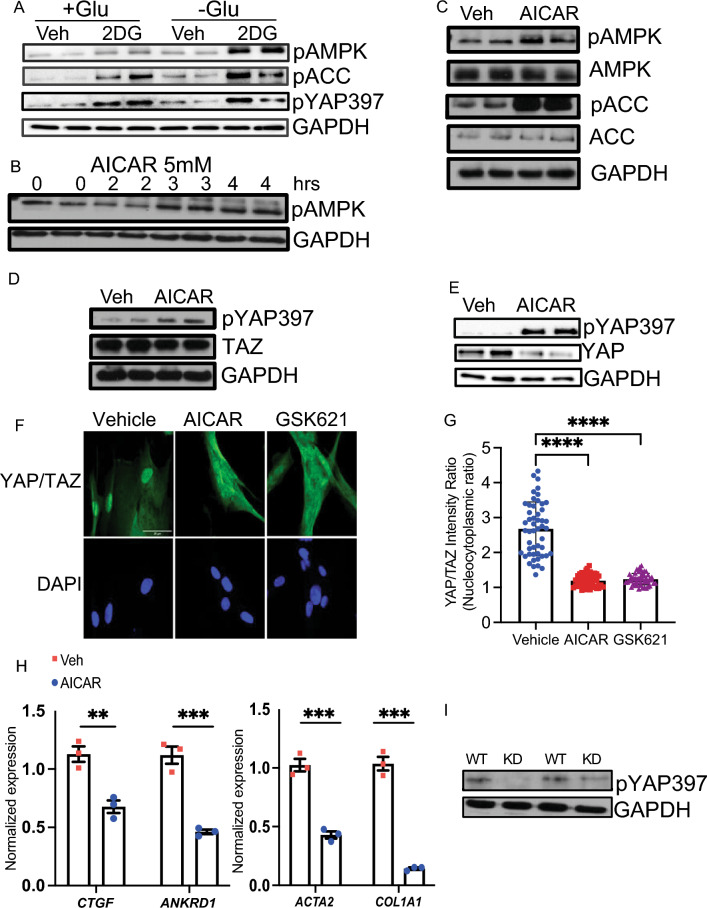
AMPK stimulation inhibits YAP/TAZ signaling to promote HSC inactivation. (**A**) HSCs were treated with 2DG (25 mM) or water vehicle in glucose-rich (25 mM) or glucose-free (0 mM) DMEM for 1 h. Expression of Phospho-AMPKα (T172), Phospho-ACC (Ser79), Phospho-YAP (Ser397) were quantified by Western blot with GAPDH as a loading control. (**B**–**E**) HSCs were treated with AICAR (5 mM) or water vehicle (Veh). Expression of Phospho-AMPKα (T172) was quantified by Western blot with GAPDH as a loading control after treatment for indicated duration in hours (hrs). (**C**) Expression of Phospho-AMPK, AMPK, Phospho-ACC, ACC, and GAPDH were quantified by Western blot after treatment for 4 h. (**D**, **E**) Expression of Phospho-YAP (Ser397), YAP, TAZ, and GAPDH were quantified by Western blot after treatment for 4 h (**D**) or 72 h (**E**). (**F**) HSCs were treated with vehicle, AICAR (5 mM), or GSK621 (30 μM) for 3 h. Immunofluorescence (IF) with anti-YAP/TAZ antibody (top, green) and DAPI (bottom, blue). Scale bar: 25 μM. (**G**) Quantification of YAP/TAZ mean fluorescence nucleocytoplasmic intensity ratio (N/C ratio) was performed for 50 cells per condition. (**H**) qRT-PCR was performed to quantify expression of the indicated genes after treatment with water vehicle or AICAR (5 mM) for 24 h. Samples were normalized to *GAPDH*. (**I**) HSCs were nucleofected with Wild type AMPKα (WT) or kinase-inactive AMPKα (KD). Expression of Phospho-YAP (Ser397) and GAPDH were quantified by Western blot after 4 h. *n* = 2 biologically independent samples and results are representative of 3 independent experiments. Data are expressed as mean ± s.e.m. Subsequent statistical analysis was performed with unpaired two-sided student t-tests or one-way ANOVA with Tukey’s method for multiple comparisons. (** *P* < 0.01, *** *P* < 0.001, **** *P* < 0.0001).

Prior studies have demonstrated a critical role of cellular energy stress on YAP/TAZ signaling^18,19^. We thus asked whether modulating energy stress levels in HSCs regulates YAP/TAZ activity. To model energy stress, we glucose-starved HSCs for one hour and added 2-deoxy-D-glucose (2DG), a nonmetabolizable glucose analog that inhibits normal glucose metabolism. Expectedly, the addition of 2DG promoted AMPK activation, as measured by increased phosphorylation of the activation loop T172 in AMPKα and increased phosphorylation of acetyl-CoA Carboxylase (ACC), an AMPK substrate, and this effect was increased in glucose-starved HSCs (Fig. 1A).

The transcriptional activity of YAP/TAZ is influenced by their localization in the nucleus or cytoplasm, which is regulated by multiple inputs including mechanical stimuli and G protein-coupled receptor signaling^20–22^. Several of these inputs are relayed through the LATS1/2 kinase cascade, where activated phospho-LATS1/2 phosphorylate YAP/TAZ on serine residues, including serine 127 (S127 in YAP; S89 in TAZ), which promotes YAP/TAZ cytoplasmic retention, and serine 397 (S397 in YAP; S311 in TAZ), which promotes YAP/TAZ degradation^23^. We observed that 2DG treatment promotes YAP S397 phosphorylation, suggesting that cellular energy stress inhibits YAP/TAZ signaling in HSCs (Fig. 1A).

To further interrogate the relationship between AMPK and YAP/TAZ signaling in HSCs, we utilized a pharmacologic activator of AMPK, 5-Aminoimidazole-4-carboxamide ribonucleotide (AICAR), which is an analog of adenosine monophosphate^24^. We performed a time-course experiment and observed that treatment with AICAR promotes phosphorylation of AMPK and ACC (Fig. 1B,C).

We next examined how AICAR modulates YAP/TAZ phosphorylation and observed that AICAR increases YAP phosphorylation at serine 397 (Fig. 1D). Phosphorylation of S397 by LATS1/2 primes for additional phosphorylation of YAP, which leads to recruitment of β-TrCP, a key adaptor of SCF E3 ubiquitin ligase. This leads to subsequent proteasomal degradation^23^. Consistently, we observed a reduction in TAZ protein levels at 4 h (Fig. 1D). Given YAP has a longer half-life compared to TAZ^5^, we treated HSCs with AICAR or vehicle for 72 h. We observed that AICAR increases YAP phosphorylation at serine 397 and reduces YAP protein levels at this time point (Fig. 1E). AICAR did not affect YAP phosphorylation at serine 127 (Fig. S1A).

We then measured the effect of AMPK activation on YAP/TAZ nuclear localization. We observed that treatment with AICAR or another AMPK activator, GSK621, promotes cytoplasmic localization and reduces the nucleocytoplasmic ratio (Fig. 1F,G). Furthermore, we observed that AICAR suppresses expression of YAP/TAZ transcriptional targets, including *CTGF* and *ANKRD1*, and markers of HSC activation and collagen production, *ACTA2* and *COL1A1* (Fig. 1H).

To support our studies using pharmacologic activators of AMPK, we expressed wild type AMPKα or the kinase-inactive AMPKα and measured the impact on YAP phosphorylation. We observed that the kinase-inactive AMPKα decreases YAP phosphorylation at serine 397 compared to wild-type AMPKa (Fig. 1I). Taken together, our studies demonstrate that AMPK activation suppresses YAP/TAZ signaling and HSC activation.

### AMPK suppression of YAP/TAZ signaling is LATS1/2-dependent

**Figure 2:**
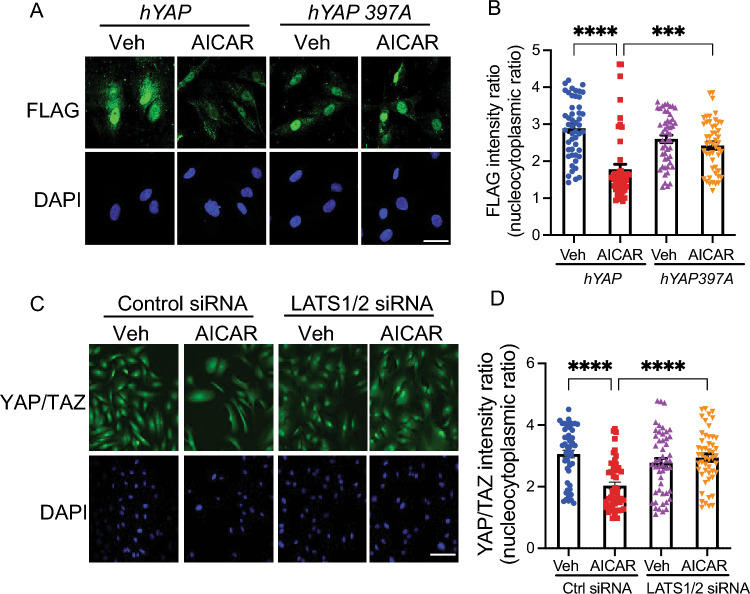
AMPK regulation of YAP/TAZ localization is mediated by LATS1/2. (**A**) HSCs were nucleofected with FLAG-tagged *hYAP* or *hYAP*^*S397A*^. After 24 h, cells were treated with water vehicle or AICAR (50 mM) for 3 h. IF with anti-FLAG antibody (top, green) and DAPI (bottom, blue). Scale bar: 10 μm. (**B**) Quantification of FLAG N/C ratio was performed for 50 cells per condition. (**C**) HSCs were transfected with nontargeting siRNAs (control) or siRNAs targeting LATS1 and LATS2. After 48 h, cells were treated with water vehicle or AICAR (50 mM) for 3 h. IF with anti-YAP/TAZ antibody (top, green) and DAPI (bottom, blue). Scale bar: 10 μm. (**D**) Quantification of YAP/TAZ N/C ratio was performed for 50 cells per condition. Results are representative of 3 independent experiments. Data are expressed as mean ± s.e.m. Subsequent statistical analysis was performed with one-way ANOVA with Tukey’s method for multiple comparisons. (*** *P* < 0.001, **** *P* < 0.0001).

The LATS1/2 kinases are the major kinases responsible for YAP/TAZ inhibition. We next interrogated whether AMPK regulation of YAP/TAZ signaling is mediated by the LATS1/2 kinases. We used a mutant allele of human *YAP*, *hYAP*^*S397A*^, which cannot be phosphorylated at serine 397 and thus cannot be degraded. We nucleofected human HSCs with control *hYAP* or *hYAP*^*S397A*^ and treated them with AICAR or vehicle. In cells over-expressing the control *hYAP*, immunostaining showed that AICAR significantly inhibits nuclear localization compared to vehicle. However, in cells expressing *hYAP*^*S397A*^, the effect of AICAR on YAP/TAZ nuclear localization was abrogated (Fig. 2A,B).

Consistently, depletion of LATS1/2 by siRNA attenuated the effect of AICAR on YAP/TAZ nuclear localization (Fig. 2C,D). These studies suggest that AICAR promotes LATS1/2-mediated YAP/TAZ phosphorylation and cytoplasmic localization, leading to suppression of YAP/TAZ signaling.

Prior studies have suggested a key role of angiomotin-like 1 (AMOTL1) in AMPK-YAP/TAZ signaling: specifically, it was demonstrated that AMPK phosphorylates and stabilizes AMOTL1 to promote LATS1/2 activity^19^. We next investigated whether AMPK activation by AICAR regulates AMOTL1 levels in HSCs. Treatment with AICAR did not alter AMOTL1 levels (Fig. S2A), suggesting that AMPK regulates LATS1/2 activity independent of AMOTL1.

### AMPK activation inhibits YAP/TAZ signaling and attenuates CCl_4_-induced hepatic fibrogenesis

To investigate the effect of AMPK on YAP/TAZ signaling in vivo, we utilized the carbon tetrachloride (CCl_4_) mouse model of fibrosis. In this model, male C57BL/6J mice (age 6 weeks, Jackson Laboratory, Bar Harbor, ME) were injected with CCl_4_ or olive oil three times weekly for 6 weeks. Beginning at week 3, mice received AICAR (350 mg/kg) or vehicle control by intraperitoneal injection three times weekly and were sacrificed at week 6. This timing was selected to measure the impact of AICAR after fibrosis has been established, which mirrors the treatment of patients with established disease. Prior studies evaluated similar dosing of AICAR in CCl_4_ and high-fat diet mouse models and demonstrated that AICAR increased AMPK activity in mouse liver tissues and was well-tolerated^25–27^. In our study, we observed that AICAR treatment reduced total body weight among mice receiving olive oil. There were no differences in body weight among mice receiving CCl_4_ (Fig. S3A). In addition, there were no differences in liver proportional weight between treatment groups (Fig. S3B).

**Figure 3:**
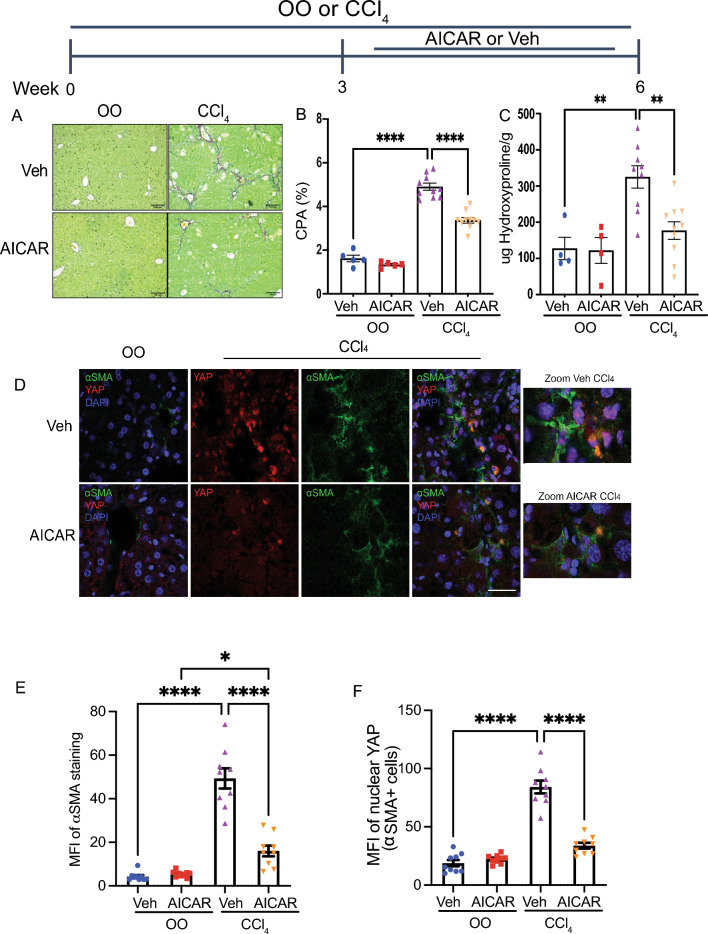
AMPK stimulation inhibits YAP/TAZ and ameliorates liver fibrosis. Male C57BL/6 J mice (age 6–8 weeks) received olive oil (OO) or CCl_4_ three times per week by IP injection for a total of 6 weeks. AICAR (350 mg/kg) or water vehicle (Veh) were concomitantly administered by IP injection three times per week for the last 3 weeks of OO or CCl_4_ treatment. (**A**) Representative images of liver tissues with Sirius-red staining. Scale bar: 100 μm. (**B**) Morphometric assessment of the collagen proportional area (CPA) on Sirius-red stained slides was performed for 5 sections per mouse measured at 10×, and the average value for each mouse is shown. (**C**) Hepatic hydroxyproline was measured in mice from each treatment group. (**D**) Representative IF images of liver tissues for YAP (red), α-SMA (*ACTA2*, green), and DAPI (blue). Scale bar: 30 μm. (**E**, **F**) Mean fluorescence intensity (MFI) was measured in a blinded fashion by ImageJ for 9 cells per mouse and 3 mice were included for each treatment group for a total of 27 cells per treatment group. We calculated the average MFI value of 3 samples taken in chronological order (ie the average of 1,2,3/4,5,6 etc.), which resulted in 3 MFI values/mouse for 3 mice per treatment group. The MFI values of 9 samples per treatment condition are displayed. MFI of α-SMA was measured within the cytoplasm, and among α-SMA-positive cells, MFI of YAP was measured within the nucleus as identified by DAPI. For collagen proportional area and hydroxyproline measurements, *n* = 5 in Veh + OO, *n* = 5 in AICAR + OO, *n* = 10 in Veh + CCl_4_, *n* = 10 in AICAR + CCl_4_. Data are expressed as mean ± s.e.m. Subsequent statistical analysis was performed with one-way ANOVA with Tukey’s method for multiple comparisons. (* *P* < 0.05, ** *P* < 0.01, **** *P* < 0.0001).

Furthermore, AICAR treatment led to attenuation of fibrosis, as evidenced by a decrease in Sirius red staining, collagen proportional area, and hydroxyproline (Fig. 3A–C). We observed that AICAR significantly decreases HSC activation as measured by αSMA immunofluorescence (Fig. 3D,E). In addition, we observed a reduction in YAP nuclear localization in HSCs among CCl_4_-treated mice receiving AICAR compared to those receiving vehicle (Fig. 3D,F), suggesting that AICAR inhibits YAP signaling in vivo. We did not observe significant differences in inflammation as measured by histologic evaluation (Fig. S3C,D).

## Discussion

End-stage liver disease affects approximately 600,000 Americans and accounts for 30,000 deaths each year^28,29^. The number of patients affected by liver disease is expected to rise with the increasing prevalence of metabolic dysfunction-associated steatotic liver disease (MASLD), which is now estimated at 30% in urban American adults^30^. Fibrosis is the common endpoint responsible for liver failure in nearly every form of chronic liver disease, yet there are a paucity of treatment options that inhibit fibrosis progression. Furthermore, hepatocellular carcinoma is a devastating disease, and 80–90% of these cases are associated with severe fibrosis^31^. Thus, there is an urgent need to identify new approaches to treat hepatic fibrosis.

Activation of hepatic stellate cells is the key step in hepatic fibrogenesis, and therapies to inactivate HSCs have potential as antifibrotic agents. The Hippo pathway and its key effectors, YAP and TAZ, play a critical role in HSC activation and hepatic fibrosis: YAP inhibition leads to HSC inactivation and hepatic fibrosis reduction^5–10^. However, since YAP/TAZ signaling confers disease-specific and cell-type dependent effects^11^, it is important to clarify regulators of YAP/TAZ in HSCs as a strategy to reduce fibrogenesis. In this study, we show that stimulation of AMPK, a central regulator of energy homeostasis, inhibits YAP/TAZ signaling in HSCs and ameliorates hepatic fibrogenesis in the setting of established fibrosis.

Prior studies have implicated AMPK in HSC activation and fibrogenesis. For example, AMPK activation has been shown to inhibit fibrogenesis in rodent models of fibrosis, including in a NASH model and a bile duct ligation model^24,32–37^. Several studies have highlighted potential mechanisms by which AMPK activation inhibits HSC activity and proliferation, such as modulation of TGFβ^24^ and nitric oxide synthase^38^. Our work demonstrates for the first time that AMPK activation in HSCs disrupts YAP/TAZ signaling, a core pathway in fibroblast activation. Furthermore, we show that AICAR inhibits YAP/TAZ in HSCs by promoting LATS1/2, the major kinase responsible for YAP/TAZ phosphorylation, which is consistent with a study by Mo et al. in mouse embryonic fibroblasts^18^. AMPK has also been shown to phosphorylate several serine residues of YAP, such as S61, S94, S366, and S463^18,39^. In this study, we chose to focus on S127 and S397 sites. Our previous study identified S397 as a critical phosphorylation site that inhibits YAP/TAZ nuclear localization and promotes their degradation in HSCs while the S127 phosphorylation was unaffected by the sphingolipid ceramide^5^. Consistent with our previous report, we found that AMPK activation by AICAR increases phosphorylation at S397 while S127 phosphorylation is unchanged.

In addition, this present study demonstrates that AMPK signaling promotes LATS1/2-mediated YAP/TAZ phosphorylation independent of AMOTL1. This suggests that LATS1/2 might be directly regulated by AMPK activation in HSCs or other kinases downstream of AMPK might be involved. Additional studies are ongoing to characterize how AMPK modulates LATS1/2 signaling in HSCs.

Our study had several limitations. For example, we employed a pharmacologic activator of AMPK, AICAR, in the CCl_4_ mouse model of fibrosis. Intraperitoneal administration of AICAR likely had effects on other cell types, including hepatocytes, which may have contributed to fibrosis attenuation. To address these limitations, studies are ongoing to measure the effect of HSC deletion of AMPK in multiple mouse models of fibrosis. Importantly, a prior study to evaluate the role of AMPK deficiency in CCl_4_-induced fibrosis was limited by inclusion of only female AMPKα1 null-mice^40^. AMPK exists as a heterotrimeric complex containing a catalytic subunit (encoded by α1 or α2), and this study did not examine the compensatory role of AMPKα2.

We selected the CCl_4_ model of hepatic fibrosis, as it is a well-established and widely accepted experimental model to study liver fibrosis^41^. It recapitulates the pattern of injury observed in patients, including hepatocyte injury, macrophage infiltration, activation, and collagen deposition in a relatively short duration of time. Studies to decipher the impact of AMPK signaling using other clinically relevant mouse models of hepatic fibrosis, including models of MASLD and alcoholic liver disease, are warranted.

In addition, we did not explore whether AMPK activation has differential effects on YAP and TAZ in HSCs. Prior studies have suggested distinct roles for YAP or TAZ in cell function: for example, Plouffe et al. observed that YAP inactivation has a greater effect on cell spreading, proliferation and migration than TAZ inactivation in HEK293A cells, though functional redundancy was also observed^42^. The literature on the potential distinct roles of YAP and TAZ in fibroblasts is limited. A study by Link et al. in lung fibroblasts demonstrated limited effects of knockdown of YAP or TAZ alone compared to combined knockdown, suggesting cooperation or redundancy of YAP and TAZ^43^. In this study, we observed that AMPK activation decreases TAZ and YAP protein levels at 4 and 72 h, respectively, consistent with the different half-lives of YAP and TAZ^5^, suggesting that AMPK signaling regulates both YAP and TAZ. Additional studies are ongoing to determine whether there is preferential regulation by AMPK activation of YAP or TAZ and whether feedback signaling occurs in HSCs.

In summary, our study has identified a novel mechanistic link between AMPK and YAP/TAZ in the context of HSC activation and hepatic fibrogenesis. Further elucidation of key regulators of AMPK-YAP/TAZ pathway in HSCs may lead to additional strategies to promote fibrosis regression.

## Methods and materials

### Cell culture studies

#### Cell culture

Adult human HSCs isolated from human liver tissue samples were obtained from ScienCell Research Laboratories. AICAR (2840, Tocris) was dissolved in water while GSK621 (S7898, Selleckchem) was dissolved in DMSO. 2DG (D8375, Sigma) was dissolved in water. Cells were grown in a humidified 5% CO_2_ atmosphere at 37 °C in Dulbecco’s Modified Eagle Medium (DMEM with 4.5 g/L glucose) with 10% fetal bovine serum (FBS), 1% Penicillin/Streptomycin (P/S), and 1% Normocin (ant-nr-1, InvivoGen). For glucose starvation experiments, glucose-free DMEM (11966025, Gibco) with 10% FBS, 1% P/S, and 1% Normocin was used.

#### Nucleofection

Nucleofection of HSCs was performed as previously described^5^. Briefly, 5.0 × 10^5 HSCs (per reaction) were resuspended in 100 μl of nucleofection buffer^42^ with 1 μg plasmid in a Nucleocuvette Vessel (Lonza). The constructs used for nucleofection: pcDNA-Flag-Yap1 (gift from Ophir Klein, Addgene plasmid #18,881), pCMV-Flag-YAP-S381A (gift from Ophir Klein, Addgene plasmid #27,377), WT AMPKα1, Kinase inactive DN mutant (gift from Kun-Liang Guan, University of California, San Diego, La Jolla, CA). Cells were nucleofected using the FF-113 program for P3 primary cells on the 4D-Nucleofector Core and X Unit system (Lonza) according to the manufacturer’s instructions. 200 μl of prewarmed media was added to the sample, which was then transferred to one well of a 12 well plate with 700 μl of prewarmed media.

#### RNA interference

siGENOME small interfering RNAs (siRNA) of targeted human LATS1 (D-0046320-01), LATS2 (D-003865-01), and non-targeting siRNA (siGENOME) (D-001210-01, D-001210-02) were obtained from Dharmacon. Cells were plated in 12-well plates (80,000 cells/well) and reverse transfected with 50 nM siRNA using DharmaFECT 1 (Dharmacon) in Dulbecco’s Modified Eagle Medium (DMEM) with 10% fetal calf serum (FCS) according to the manufacturer’s instructions^5^.

#### Immunofluorescence

HSCs were fixed with 4% paraformaldehyde (Electron Microscopy Sciences) in Phosphate-buffered saline (PBS) for 10 min and then blocked in 10% donkey serum (Jackson Immunoresearch) and 0.1% Triton-100x (BioRad) for 1 h at room temperature (RT). Cells were incubated with primary antibody diluted in blocking buffer overnight at 4 °C. Primary antibodies used were anti-YAP/TAZ (1:500, sc-101199, Santa Cruz) and anti-DYKDDDDK (1:500, PA1-984B, ThermoFisher Scientific). Cells were washed with PBS three times and incubated with Alexa 488 (1:400, ab150073 and ab150105, Abcam) and DAPI (2 μg/mL, D1306, ThermoFisher Scientific) for 1 h at RT. Cells were imaged on the Zeiss LSM 780 confocal microscope at 63 × magnification. In each experiment, laser intensity, background level, contrast, and electronic zoom size were collected at the same level^5^.

#### Measurement of nuclear/cytoplasmic fluorescence intensity ratio

Mean fluorescence intensity (MFI) was measured for 50 cells/per condition from 3 technical replicates in a blinded fashion by ImageJ. One MFI was taken within the nucleus, as identified by DAPI staining, and one within the cytoplasm, and a nuclear to cytoplasmic ratio was generated per cell^5,44^.

#### Western blotting

Western blotting was performed as previously described^5^. Primary antibodies used were: YAP (1:500, 14074S, Cell signaling)^5^, phospho-YAP Ser397^5^ (1:1000, 13619S, Cell Signaling), phospho-AMPKα (Thr172) (1: 500, 2535S, Cell Signaling), AMPKα (1:500, 5832S, Cell Signaling), Phospho-Acetyl-CoA Carboxylase (Ser79) (1:1000, 11,818, Cell Signaling), Acetyl-CoA Carboxylase (1:1000, 3662S, Cell Signaling) and GAPDH^5^ (1:2000, 2118S, Cell Signaling). The secondary antibody used was anti-rabbit IgG, HRP (horseradish peroxidase) (1:1000, 7074 V, Cell Signaling). Quantitative densitometry was performed using ImageJ software.

#### Quantitative PCR analysis

RNA was isolated from HSCs using Trizol Reagent (Life Technologies)^5^. RNA was reverse transcribed with iScript (Bio-Rad). Power SYBR Green master mix (Life Technologies) was used for quantification of cDNA on a CFX384 Real-Time System (Bio-Rad). The primers are- forward GAPDH, 5′ -ACAACTTTGGTATCGTGGAAGG-3′, reverse GAPDH, 5′-GCCATCACGCCACAGTTTC-3′, forward COL1A1, 5′-CAGGCTGGTGTGATGGGATT-3′, reverse COL1A1, 5′-AGCTCCAGCCTCTCCATCTT-3′, forward ACTA2, 5′-TCCCATCCATTGTGGGACGT-3′, reverse ACTA2, 5′-TTGCTCTGTGCTTCGTCACC-3′, forward ANKRD1, 5′-GGAGCCCAGATCGAATTCCG-3′, reverse ANKRD1, 5′-CGGGCGCTAATTTTTGCTCC-3′, forward CTGF, 5′-GACGAGCCCAAGGACCAAAC-3′, reverse CTGF, 5′-TCATAGTTGGGTCTGGGCCA-3’. The 2−ΔCT method was used for relative quantification of mRNA with normalization to GAPDH.

### Animal studies

The study is reported in accordance with ARRIVE guidelines.

#### CCl_4_ injection and AICAR treatment

Animal experiments were approved by the Institutional Animal Care and Use Committee of the University of California, San Francisco. All animals received humane care according to the criteria outlined in the Guide for the Care and Use of Laboratory Animals of the National Academy of Sciences.

For the therapeutic AICAR experiment, male C57BL/6J mice (age 6 weeks, Jackson Laboratory, Bar Harbor, ME) were injected with 0.1 cc of a 6.66% solution of CCl4 (Sigma) in olive oil or olive oil alone 3 days/week for 6 weeks. Mice were concomitantly treated with 350 mg/kg AICAR (soluble in water, ab146594, Abcam) or water vehicle 3 days/week by IP for the last 3 weeks of olive oil or CCl_4_ treatment.

All animals were anesthetized and sedated with ketamine and xylazine at the time of sacrifice. A terminal blood collection was performed by cardiac puncture. Livers were weighed and subsequently snap-frozen or fixed in formalin or 4% paraformaldehyde (PFA)^5^.

#### Histology, immunohistochemistry, immunofluorescence

Formalin-fixed samples were embedded in paraffin, cut in 5 μm thick sections, and stained with Sirius red. Slides were evaluated by a blinded expert UCSF liver pathologist (AM) for lobular inflammation. The collagen proportional area (CPA) was morphometrically quantified on Sirius red-stained sections with image processing software (Image J, NIH)^5^.

For immunofluorescence, PFA fixed samples were embedded in optimal cutting temperature (OCT) and sectioned as previously described^5^. The samples were blocked with 0.1% Triton-100x (BioRad), 10% Donkey Serum (Jackson ImmunoResearch), and 3% bovine serum albumin (BSA) in tris-buffered saline (TBS) and tween 20 (TBST). Samples were incubated with YAP (1:100, 14074T, Cell Signaling) overnight at 4 °C. The following day, after three washes with PBS, the samples were incubated with DAPI (2 μg/mL, D1306, ThermoFisher Scientific), Alexa 488 (1:200, ab150073, Abcam), and αSMA-Cy3 (1:200, C6198, Sigma Aldrich) at RT for 1 h. Slides were imaged on the Zeiss LSM 780 confocal microscope at 63 × magnification. In each experiment, laser intensity, background level, contrast, and electronic zoom size were collected at the same level.

#### Hydroxyproline assay

Snap frozen liver tissue (~ 100 mg) was thawed and homogenized in 1 ml water. Next, 125ul of 50% Trichloroacetic acid (TCA, T6399, Sigma Aldrich) was added to the homogenate and incubated on ice for 20 min. After incubation, samples were centrifuged at 1000 RPM for 5 min at 4 °C. The pellets were hydrolyzed in 12 N HCl overnight at 110 °C. The dried pellets were reconstituted in water and incubated for 6 hrs at room temperature, and incubated in Chloramine T solution (1.4% chloramine T, 402869, Sigma Aldrich in 0.5 M Sodium Acetate, S2889, Sigma Aldrich and 10% isopropanol, I9516, Sigma Aldrich) for 20 min at room temperature. Finally, 500ul of Ehrlichs solution (1 M p-Dimethylaminobenzaldehyde, 156477, Sigma Aldrich in 70% isopropanol and 30% perchloric acid, 311421, Sigma Aldrich) was added for 40 min at 65 °C. The absorbance of each sample was measured at 550 nm.

#### Statistics

All data are represented as the mean of at least 2 independent experiments ± SEM. Statistical analysis was performed with GraphPad Prism 9.3.1 in either unpaired two-sided student t-tests or one-way ANOVA with Tukey’s method for multiple comparisons. Statistical significance was defined as *P* < 0.05. All experiments were performed a minimum of two times unless otherwise stated.

### Supplementary Information

Supplementary Figures.

## Data Availability

The data generated during the current study are available from the corresponding author on reasonable request.
