# Supplementary material for: AMPK stimulation inhibits YAP/TAZ signaling to ameliorate hepatic fibrosis
Source: Sci Rep. 2024 Mar 3;14:5205. doi: 10.1038/s41598-024-55764-5 (PMC10909858; doi:10.1038/s41598-024-55764-5)
Supplement: Supplementary file 1 — Supplementary Figures. [file 41598_2024_55764_MOESM1_ESM.pdf]

AMPK Stimulation Inhibits YAP/TAZ Signaling to Ameliorate Hepatic Fibrosis

Mahbubul H. Shihan[1*], Sachin Sharma[1*], Carson Cable[1], Vijaya Prathigudupu[1], Alina Chen[1], Aras N. Mattis[2,3], and Jennifer Y. Chen**[1, 3]

1. Department of Medicine, University of California, San Francisco, San Francisco, CA 94115.

2. Department of Pathology, University of California, San Francisco, San Francisco, CA 94143

3. The Liver Center, Department of Medicine, University of California, San Francisco, San Francisco, CA 94143.

*These authors have contributed equally to this work and share first authorship.

**To whom all correspondence should be addressed. Email: Jennifer.Chen4@ucsf.edu

Supplemental Figures and Legends

Supplemental Figure 1

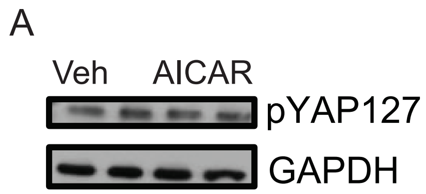

Supplemental Fig. 1. AMPK activation by AICAR does not affect YAP phosphorylation at Serine 127. HSCs were treated with AICAR (5 mM) or water vehicle (Veh) for 4 hours. **(A)** Expression of Phospho-YAP (Ser127) was quantified by Western blot with GAPDH as a loading control.

Supplemental Figure 2

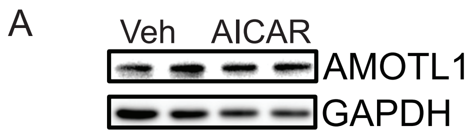

Supplemental Fig. 2. AMPK activation by AICAR does not affect AMOTL1 protein levels. HSCs were treated with AICAR (5 mM) or water vehicle (Veh) for 3 hours. **(A)** Expression of AMOTL1 was quantified by Western blot with GAPDH as a loading control.

Supplemental Figure 3

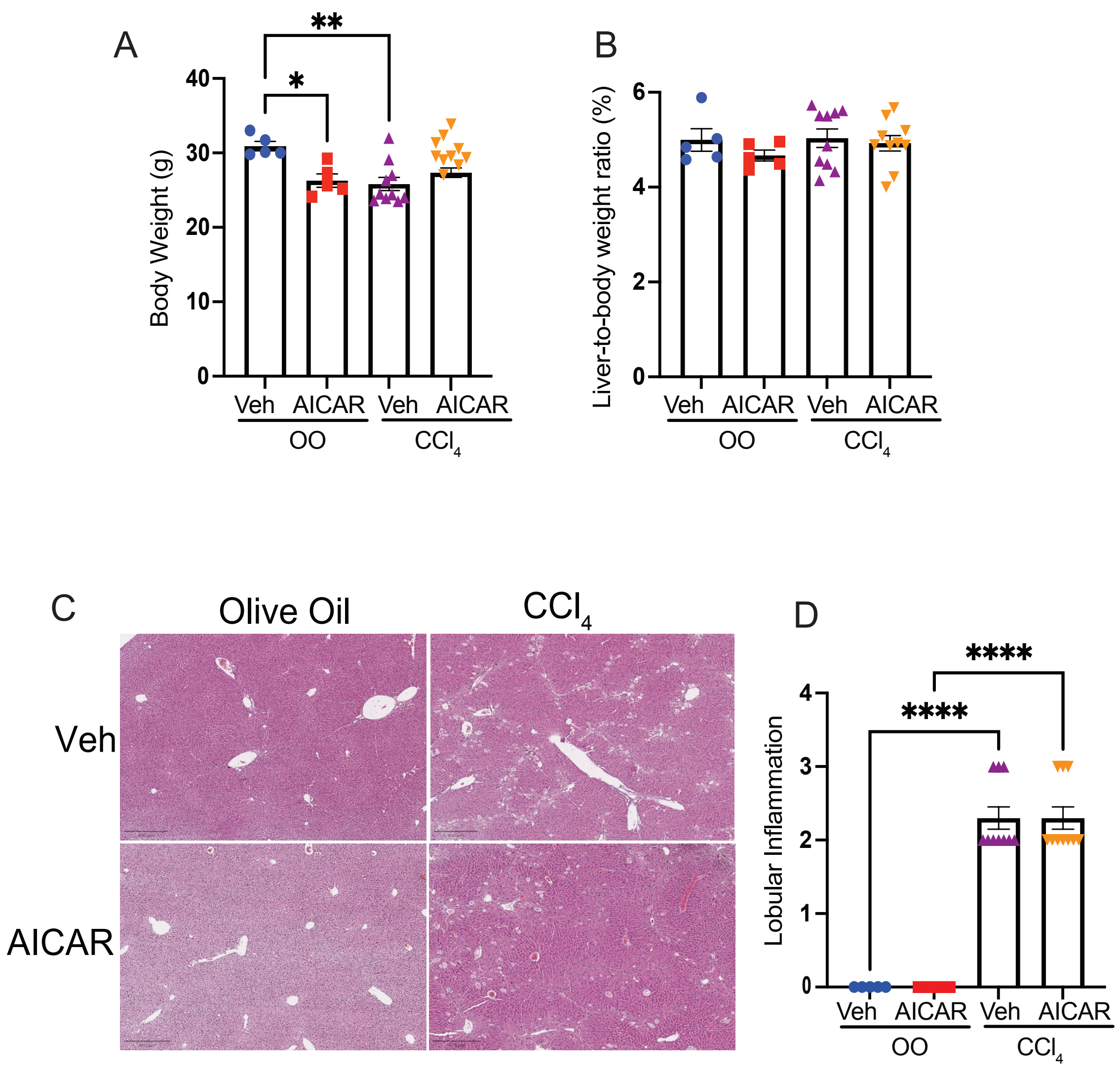

Supplemental Fig. 3. AICAR treatment does not modulate liver proportional weight in the CCl_4 mouse model. Male C57BL/6J mice (age 6-8 weeks) were injected with olive oil (OO) or CCl_4 three times a week for a total of 6 weeks. AICAR (350 mg/kg) or water vehicle (Veh) were concomitantly administered by IP injection three times per week for the last 3 weeks of OO or CCl_4 treatment. **(A)** Body weight measured in grams. **(B)** Liver weight-to-body ratio. **(C)** Representative images of liver sections with hematoxylin and eosin staining. Scale bar, 400 μm. **(D)** Histologic evaluation of lobular inflammation performed by blinded pathologist. $n=5$ in Veh+OO, $n=5$ in AICAR+OO, $n=10$ in Veh+CCl_4, $n=10$ in AICAR+CCl_4. Data are expressed as mean ± s.e.m. Subsequent statistical analysis was performed with one-way ANOVA with Tukey's method for multiple comparisons. (* $P < 0.05$, ** $P < 0.01$, **** $P < 0.0001$)
